# Effects of apoE genotype on macrophage inflammation and heme oxygenase-1 expression

**DOI:** 10.1016/j.bbrc.2007.03.150

**Published:** 2007-05-25

**Authors:** Laia Jofre-Monseny, Agnieszka Loboda, Anika E. Wagner, Patricia Huebbe, Christine Boesch-Saadatmandi, Alicja Jozkowicz, Anne-Marie Minihane, Jozef Dulak, Gerald Rimbach

**Affiliations:** a Institute of Human Nutrition and Food Science, Christian Albrechts University of Kiel, Hermann-Rodewald-Strasse 6, 24098 Kiel, Germany; b Department of Medical Biotechnology, Faculty of Biochemistry, Biophysics and Biotechnology, Jagiellonian University, Gronostajowa 7, 30-387 Krakow, Poland; c School of Chemistry, Food Biosciences and Pharmacy, University of Reading, Reading RG6 6AP, UK

**Keywords:** apoE genotype, Macrophage, Cytokines, Nuclear factor κB, Heme oxygenase-1, Tumour necrosis factor α, Inflammation, Oxidative stress, Redox signalling

## Abstract

In order to gain a more comprehensive understanding of the aetiology of apolipoprotein E4 genotype-cardiovascular disease (CVD) associations, the impact of the apoE genotype on the macrophage inflammatory response was examined. The murine monocyte–macrophage cell line (RAW 264.7) stably transfected to produce equal amounts of human apoE3 or apoE4 was used. Following LPS stimulation, apoE4-macrophages showed higher and lower concentrations of tumour necrosis factor alpha (pro-inflammatory) and interleukin 10 (anti-inflammatory), respectively, both at mRNA and protein levels. In addition, increased expression of heme oxygenase-1 (a stress-induced anti-inflammatory protein) was observed in the apoE4-cells. Furthermore, in apoE4-macrophages, an enhanced transactivation of the key redox sensitive transcription factor NF-κB was shown. Current data indicate that apoE4 macrophages have an altered inflammatory response, which may contribute to the higher CVD risk observed in apoE4 carriers.

Apolipoprotein E (apoE) is a polymorphic multifunctional protein with three common isoforms in humans (E2, E3, and E4). ApoE3 is the wild-type and most common isoform, while apoE4 carriers account for about 25% of the Caucasian population [1]. Presence of the apoE4 allele is associated with a 40–50% higher risk of cardiovascular disease (CVD) [2] and apoE4 is the major known genetic risk factor for maturity-onset Alzheimer’s disease (AD) [3].

Although apoE4 is strongly linked to both diseases, the molecular basis of these associations remains uncertain. Traditionally, the differential risk has been attributed to the increased low density lipoprotein cholesterol (∼8%) observed in apoE4 carriers, but it is becoming increasingly evident that this alone cannot explain the disease differential [4].

ApoE is not only synthesised by the liver, but also in the brain and by resident macrophages [5] in the atherosclerotic wall, where it exerts atheroprotective actions, independent of its role in lipid metabolism [6]. These localised functions include regulation of smooth muscle cell migration and proliferation [7], inhibition of adhesion molecule expression in endothelial cells [8] and inhibition of platelet aggregation [9]. In addition to its paracrine effect on surrounding cells, apoE has also been shown to impact on macrophage function by promoting cholesterol efflux [10] and modulating NO production [11]. Inflammation and oxidative stress are key features of the pathology of atherosclerosis and AD. A limited number of studies, which have largely focussed on brain biology and neurodegeneration [12,13] have reported on the immuno-modulatory properties of apoE and its impact on inflammatory mediators [14]. However, little is known about the possible role of apoE genotype as a mediator of the macrophage innate immune and inflammatory responses in relation to CVD.

Using a murine macrophage cell line, which has been stably transfected with human apoE3 or apoE4, we have recently showed that apoE isoform affects macrophage oxidative status [15], and now we hypothesise that this may be accompanied by an altered inflammatory response. Furthermore, the impact of genotype on the activity of the transcription factor nuclear factor κB (NF-κB), which is known to play a major role in modulating the inflammatory response, will be presented.

## Methods

*Cell culture*. RAW 264.7 murine macrophage cell lines, stably transfected with either human apoE3 or apoE4 were kindly given by Dr. B. Pitas (Gladstone Institute, UCSF, USA). Cells were genotyped for human apoE3 and apoE4 by the method of Hixson and Vernier [16] and apoE concentrations were measured in supernatants to ensure that secreted levels in 24 h were physiological and comparable among the two clones. Mean (SEM) levels of 1.38 (0.38) and 1.36 (0.38) μg/mg cell protein were secreted in 24 h by apoE3 and apoE4 cells, respectively, as has been previously reported [15]. Cells were cultured in Dulbecco’s modified Eagle’s medium supplemented with 10% foetal bovine serum, 4 mM l-glutamine, 100 U/ml penicillin and 100 μg/ml streptomycin and 100 μg/ml G-418. Macrophages were grown in a humidified incubator at 37 °C and 5% CO_2_. Cells were incubated with lipopolysacharide (LPS) (*Salmonella enteriditis*, Sigma–Aldrich, Taufkirchen, Germany) for different time-periods depending on the outcome of interest.

*Cytokine levels*. Cells were stimulated with increasing concentrations of LPS (0–10 μg/ml) for 4 h. Supernatants were collected for analysis 20 h later. Cytokine concentrations were measured using commercial ELISA kits according to the manufacturer’s instructions. Tumour necrosis factor alpha (TNFα) was determined by the Quantikine^®^ mouse TNFα kit (R&D Systems, Wiesbaden, Germany), Interleukin (IL) 6 and 10 were measured using the Mouse Biotrak ELISA systems (Amersham Biosciences, Freiburg, Germany), and IL1β and macrophage inflammatory protein-1alpha (MIP1α) were determined with the mouse RayBio^®^ ELISA kits from Ray Biotech (Norcross, USA). Values were normalised for total cell protein which was determined using the BCA assay (Pierce Biotechnology, Rockford, USA).

*Cytokine and heme oxygenase-1 (HO-1) mRNA levels*. Cells were stimulated with LPS (1 μg/ml) for 1 h to determine TNFα mRNA levels and for 6 h to determine mRNA levels for IL1β, IL6, IL10, and MIP1α. The time-points were chosen on the basis of maximum mRNA expression for each cytokine in a time-course experiment (data not shown). Total RNA was isolated with the RNeasy Mini Kit (Qiagen, Hilden, Germany). One step RT-PCR was carried out using the QuantiTect^®^SYBR^®^Green RT-PCR kit (Qiagen) according to supplier instructions. For HO-1, cells were stimulated with LPS (1 μg/ml) for 24 h and RNA was isolated by acid guanidinium thiocyanate–phenol–chloroform extraction and reverse transcription was carried out with oligo-dT primers for 1 h at 42 °C using MMLV reverse transcriptase, according to the manufacturer’s instructions (Promega, Madison, WI, USA). Real-time RT-PCR was performed with the SYBR^®^ Green qPCR Kit (Finnzymes, Espoo, Finland). For all reactions, the Rotor Gene RG-3000 (Corbett Research) cycler was used and relative quantification of gene expression was calculated based on the 2^−ΔΔCt^ method (β-actin or elongation factor 2 were used as housekeeping genes). Primers and cycling conditions are shown in Table 1.

*Transcription factor activity*. The NF-κB-secretory alkaline phosphatase (NF-κB-SEAP) (Clontech, BD Biosciences, Palo Alto, USA) reporter construct was used to measure the binding of transcription factors to the κ enhancer, and the activation of this pathway. Cells growing in 24 well plates were transiently transfected with 0.5 μg of the vector by SuperFect^®^ transfection Reagent (Qiagen) according to the manufacturer’s protocol. Twenty-four hours later, cells were stimulated with varying concentrations of LPS (0–1 μg/ml). At 6, 12, and 24 h, the cell culture media was removed and stored for analysis. The chemiluminescent SEAP assay (Clontech) was carried out as has been previously described [17]. Values were normalised for total cell protein determined by the BCA assay (Sigma).

*Western blot analysis for heme oxygenase-1 (HO-1)*. Total cellular protein was isolated using ice-cold lysis buffer (1× PBS, 10 mM phenylmethylsulfonyl fluoride (PMSF), 10 mM leupeptine, 10 mM aprotinin and 1% Triton X-100). Samples were centrifuged (10 min, 8000*g*, 4 °C) and clear supernatants were collected. Twenty-five micrograms of protein was loaded on 12% SDS–PAGE gel followed by transfer to nitrocellulose membrane PROTRAN (Perkin-Elmer Life Sciences, Boston, USA). Following overnight blocking (4 °C in 5% non-fat milk), membranes were probed with polyclonal antibodies against HO-1 (Stressgen Biotech, Canada) and monoclonal antibodies against α-tubulin (both diluted 1:1000 in TTBS with 3% albumin) at room temperature for 1.5 h followed by anti-rabbit HRP-linked secondary antibodies (Cell Signalling Technology, USA) (1:10,000 in TTBS with 3% albumin) for 40 min at room temperature. Blots were developed with SuperSignal West Pico Chemiluminescent Substrate (Pierce Biotechnology) according to the manufacturer’s instructions.

*Statistical analysis*. Statistical calculations were conducted with SPSS Version 13.0. *T*-Tests (for independent samples) were performed to compare the outcomes between apoE3 and apoE4 cells. In the absence of normal distributed data, Mann–Whitney *U*-test was used. Results are expressed as means ± SEM and significance was accepted at *P* < 0.05.

## Results

### Cytokine protein and mRNA levels

**Fig. 1:**
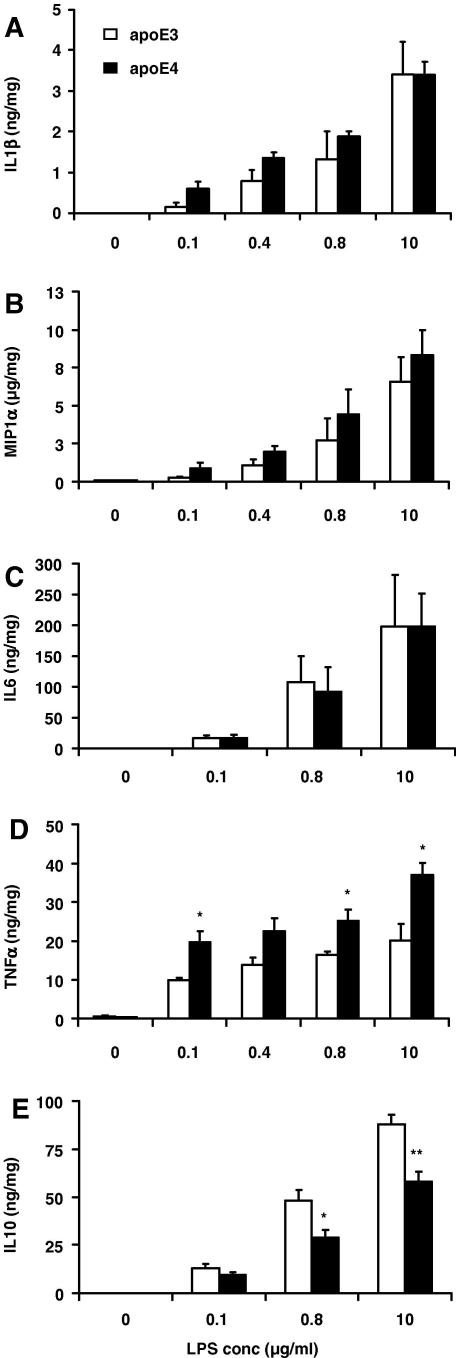
Cytokine production in RAW 264.7-apoE3 and -apoE4 following stimulation with increasing concentrations of LPS (0–10 μg/ml) for 4 h. Supernatants were collected for ELISA analysis 20 h later. (A) IL1β, (B) MIP1α, (C) IL6, (D) TNFα, (E) IL10. Data are expressed as means ± SEM of three independent experiments performed in duplicate. ^∗^*P* < 0.05, ^∗∗^*P* < 0.01, comparing E3- vs. E4-cells at each LPS concentration.

Stimulation of cells with increasing concentrations of LPS (0–10 μg/ml) resulted in a dose–response accumulation of cytokines in the cell culture media of RAW 264.7-apoE3 and -apoE4 cells (Fig. 1). In the non stimulated cells, levels of cytokines were under the limit of detection. In the cases of IL1β and MIP1α (Fig. 1A and B), there was a tendency for apoE4 expressing cells to secrete higher levels of cytokines at the majority of LPS concentrations tested, although the inter-group differences did not reach statistical significance (Fig. 1A and B). In addition, no genotype mediated differences in the IL6 concentrations detected in the media were observed (Fig. 1C). In contrast apoE4-macrophages produced 99%, 62%, 54%, and 83% more TNFα than E3 cells when stimulated at 0.1, 0.4, 0.8, and 10 μg/ml LPS (Fig. 1D). Furthermore, when IL10, an anti-inflammatory cytokine, was measured in the culture media, it was observed that apoE4-cells secreted lower concentrations of the cytokine, at all three LPS concentrations tested, with the differences reaching significance at the higher LPS concentrations (0.8 and 10 μg/ml) (Fig. 1E).

**Fig. 2:**
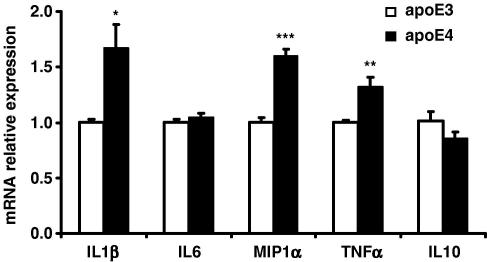
Cytokine mRNA levels measured using reverse transcription real-time PCR in RAW 264.7-apoE3 and -apoE4 following stimulation with LPS (1 μg/ml) for 6 h or 1 h (TNFα). Results are calculated with the 2^−ΔΔCt^ method and data are expressed as means ± SEM of three independent experiments performed in duplicate. ^∗^*P* < 0.05, ^∗∗^*P* < 0.01, ^∗∗∗^*P* < 0.001, comparing E3- vs. E4-cells at each LPS concentration.

To examine whether the differences in cytokine accumulation in the cell culture media were associated with differences in cytokine gene expression, the mRNA levels were measured by quantitative reverse transcription PCR analysis. Gene expression profiles revealed to be comparable to the apoE genotype mediated differences in the cytokine levels in the media, with 68%, 60% and 32% higher levels of mRNA for IL1β, MIP1α, and TNFα, respectively observed in the apoE4 transfected cells (Fig. 2). No differences could be observed in the mRNA levels of IL6 between genotypes and similar to the cytokine accumulation, IL10 mRNA levels, although not statistically different were ∼15% lower in the apoE4 cells.

### NF-κB promoter activity

**Fig. 3:**
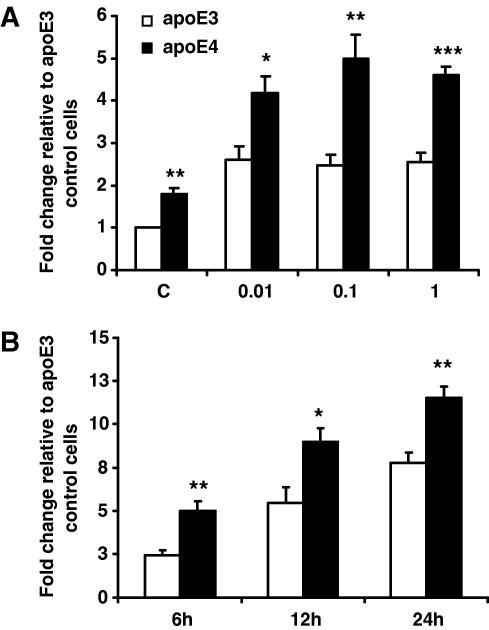
(A) NF-κB activity detected with alkaline phosphatase reporter gene assay in RAW 264.7-apoE3 and -apoE4 following stimulation with increasing concentrations of LPS (0–1 μg/ml) for 6 h. (B) NF-κB activity following stimulation with LPS (0.1 μg/ml) for 6, 12, and 24 h. Results are calculated as chemiluminescence units corrected for total protein, and as fold change of apoE3 controls. Data are expressed as means ± SEM of three independent experiments performed in duplicate. ^∗^*P* < 0.05, ^∗∗^*P* < 0. 01, ^∗∗∗^*P* < 0.001 comparing E3- vs. E4-cells at each LPS concentration; (C) control non-stimulated cells.

Our results demonstrate stronger NF-κB pathway activation in the apoE4- versus the apoE3-macrophages (Fig. 3A). In non-stimulated cells, NF-κB activity was 80% higher in apoE4 cells. Following stimulation for 6 h at different LPS concentrations, NF-κB activity augmented in both cell lines. In apoE3 expressing macrophages, NF-κB activity increased ∼2.5-fold (*P* < 0.005) at a concentration of 0.01 μg/ml, with no further increase evident with increasing concentrations of LPS. In E4 cells, a higher activation of NF-κB (∼4.2-fold change, *P* < 0.005) was evident in cells stimulated with 0.01 μg/ml LPS, with a maximum ∼5-fold increase relative to apoE3 controls evident following stimulation with 0.1 μg/ml LPS (Fig. 3A). Comparable differences between genotypes on NF-κB activity were observed at 12 and 24 h, with a time-dependent accumulation of alkaline phosphatase in the cell culture media evident after LPS (0.1 μg/ml) stimulation, with 100%, 64%, and 48% differences between genotypes evident at 6, 12, and 24 h, respectively (Fig. 3B).

### Heme oxygenase-1 (HO-1) protein and mRNA expression

**Fig. 4:**
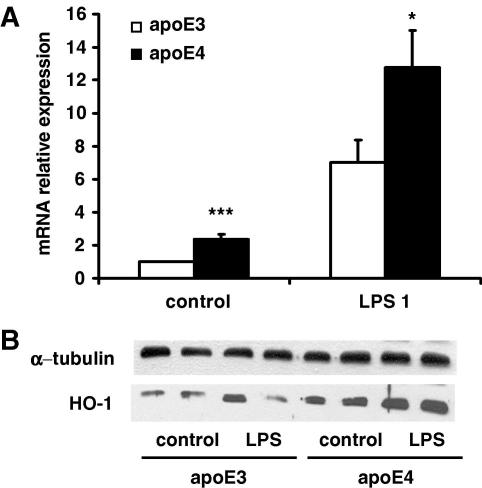
(A) HO-1 mRNA levels detected with reverse transcription real-time PCR following stimulation with LPS (1 μg/ml) for 24 h. Results are calculated with the 2^−ΔΔCt^ method and data is expressed as mean ± SEM of four independent experiments performed in duplicate. ^∗^*P* < 0.05, ^∗∗∗^*P* < 0.001, comparing E3- vs. E4-cells at each LPS concentration. (B) HO-1 levels as determined by Western blotting in relation to α-tubulin in RAW 264.7-apoE3 and -apoE4 under baseline conditions (control) and following stimulation with LPS (1 μg/ml) for 24 h.

Western blotting analysis showed higher HO-1 protein concentrations in apoE4 macrophages in both untreated (controls) and LPS (1 μg/ml) treated cells (Fig. 4B). The gene expression profiles were consistent with protein levels, with control and LPS stimulated cells expressing apoE4, producing 235% and 180% higher HO-1 mRNA relative to apoE3-macrophages (Fig. 4A).

## Discussion

We have previously reported a higher circulating [18] and macrophage [15] oxidative stress status associated with the E4 allele. Here, we extend these findings by reporting on an impact of apoE genotype on the inflammatory component of the innate immune response.

A murine monocyte-macrophage cell line (RAW 264.7) stably transfected to express either human apoE3 or apoE4 at similar concentrations and within the physiological range was used, so that the isoform-effects observed were apoE concentration-independent. In addition, LPS, a Toll-like receptor 4 (TLR 4) ligand that triggers cytokine expression by activation of a signalling cascade, was applied to investigate innate immune response, given that it is a commonly used approach, and that TLR4 activation is regarded to be relevant in the pathogenesis of atherosclerosis [19].

Following LPS stimulation, higher mRNA levels of the pro-inflammatory cytokines TNFα and, IL1β, and the chemokine, MIP1α, with a trend towards lower levels of the anti-inflammatory cytokine IL10 were evident in the apoE4 cell line. No differences were observed for IL6, which is known to act as both a pro- and anti-inflammatory mediator. Furthermore, these changes were reflected by the protein levels in the medium with the greatest differences evident for TNFα and IL10.

TNFα is pleiotropic and one of the most important pro-inflammatory and immuno-modulatory cytokines involved in the process of atherogenesis. For instance, TNFα participates in the recruitment and activation of inflammatory cells into the vessel wall by promoting matrix degradation [20] and by enhancing expression of adhesion molecules on endothelial cells [21]. In contrast, IL10 is a potent anti-inflammatory cytokine, which affects several signalling pathways and destabilizes the mRNA of pro-inflammatory genes, to contribute in the resolution of the inflammatory process [22]. Whether the higher levels of TNFα and IL1β observed in apoE4 macrophages were partly due to decreased levels of IL10 or due to other mechanisms cannot be concluded. The increased pro-inflammatory cytokine response observed in our apoE4 monocyte-macrophage cell model is in agreement with the limited amount of data available from other authors, which have examined associations between apoE genotype and inflammation in the brain. By using transgenic mice expressing human apoE3 or apoE4, Lynch et al. [14] determined that apoE4 mice showed elevated systemic and brain pro-inflammatory cytokines following intravenous administration of LPS, and Ophir et al. [12] demonstrated that the expression of inflammation genes was higher and more prolongated in the brains of apoE4 following intracerebroventricular injection of LPS. Furthermore, Maezawa et al. demonstrated that the isoform-specific patterns in cytokine production (apoE4 > apoE3 > apoE2) was specific of microglia and could not be observed in astroglia cultures [13,23]. This is particularly relevant to our studies given that microglia share many functional characteristics with macrophages. In addition to the increased pro-inflammatory response, our findings reveal that apoE4 macrophages produce decreased amounts of IL10. A recent study by Tziakas et al. [24] shows, in accordance with our results, that apoE4 carriers with acute coronary syndrome and chronic stable angina patients have lower circulating levels of IL10 than the non-apoE4 patients. Therefore taken together with the previous findings, our data support the hypothesis, that apoE4 carriers may show an “inflammatory imbalance” between pro- and anti-inflammatory mediators [24].

The regulation of cytokine production is highly complex, but the NF-κB signalling pathway is considered a key element, and is the most prominent and best characterised signal transduction pathway in TLR-mediated inflammation. In addition, the promoter regions of the cytokines presented in the current paper have putative κB binding sites (see http://people.bu.edu/Gilmore/nf-kb/). NF-κB is a redox sensitive transcription factor, and therefore it was hypothesised that the increased oxidative stress observed in the apoE4 macrophages could contribute to a higher cytokine production by enhancing the activation of NF-κB. Here, we demonstrate through a reporter gene assay, that the activity of NF-κB under basal conditions is higher in apoE4 than -E3 macrophages, and that upon stimulation with LPS, NF-κB activity increases in both cell lines, but the response is more augmented in the apoE4 macrophages. Again, this is in accordance with the results of Ophir et al. [12], who by means of microarray analysis, determined that the genes that were more differentially expressed between genotypes in the brain, where NF-κB-regulated and showed that NF-κB activation was more pronounced in the microglia of apoE4 versus apoE3 mice.

Under stress situations, the integrity of the vascular wall is maintained by several protective mechanisms, which in addition to anti-inflammatory cytokines include other proteins such as inducible heme oxygenase-1 (HO-1). HO-1 catalyses the rate limiting reaction in the degradation of heme, to yield biliverdin (with radical scavenging properties), carbon monoxide (a vascular modulator), and iron [25]. HO-1 is expressed in response to oxidative stress, and has been shown to be upregulated in atherosclerotic plaques [26] and in AD lesions [27], and attenuate inflammation and the growth of atherosclerotic plaques in transgenic mice models [28,29]. However, so far the production and role of HO-1 has not been investigated in relation to the human apoE polymorphism. Here, for the first time, we show that HO-1 levels are significantly increased in apoE4-macrophages. ApoE4-macrophages demonstrated increased levels of HO-1 under baseline conditions, and a stronger up-regulation of HO-1 at the mRNA and protein levels following LPS application.

Whether HO-1 exerts anti-inflammatory effects in apoE4 macrophages cannot be concluded from the current study. Although the inflammatory response was apparently aggravated in apoE4 cells, we cannot discard the possibility that this could be even more exacerbated without the up-regulation of HO-1 observed. Hence, one may consider, that induction of HO-1 by apoE4 represents a stress-induced protective response.

The current study is suggestive that an impact of apoE genotype on the monocyte inflammatory response may contribute to the higher CVD and AD risk observed in humans with an apoE4 genotype. However, further clarification of the molecular mechanisms, the complexity of apoE4–HO-1 interactions, as well as the impact of apoE genotype on inflammation using *in vivo* animal models and human trials is needed.

## Figures and Tables

**Table 1:** PCR primers and conditions

	Sequence (5′–3′)	Annealing *T*
β-Actin	F: GACAGGATGCAGAAGGAGATTACT	55
	R: TGATCCACATCTGCTGGAAGGT	
		
TNFα	F: CATCTTCTCAAAATTCGAGTGACAA	55
	R: TGGGAGTAGACAAGGTACAACCC	
		
IL1β	F: CAACCAACAAGTGATATTCTCCATG	55
	R: GATCCACACTCTCCAGCTGCA	
		
IL6	F: CTGCAAGAGACTTCCATCCAGTT	60
	R: GAAGTAGGGAAGGCCGTGG	
		
MIP1-α	F: CCTCTGTCACCTGCTCAACA	55
	R: GATGAATTGGCGTGGAATCT	
		
IL10	F: GGTTGCCAAGCCTTATCGGA	60
	R: ACCTGCTCCACTGCCTTGCT	
		
EF2	F: GCGGTCAGCACAATGGCATA	58
	R: GACATCACCAAGGGTGTGCAG	
		
HO-1	F: GTGGAGACGCTTTACGTAGTGC	58
	R: GACATCACCAAGGGTGTGCAG	

*Note.* F, forward primer; R, reverse primer; IL, interleukin; TNFα, tumour necrosis factor α; MIP1α, macrophage inflammatory protein 1 α; EF2, elongation factor 2; HO-1, heme oxygenase-1.
